# TNF-α Mediates PKCδ/JNK1/2/c-Jun-Dependent Monocyte Adhesion via ICAM-1 Induction in Human Retinal Pigment Epithelial Cells

**DOI:** 10.1371/journal.pone.0117911

**Published:** 2015-02-12

**Authors:** I-Ta Lee, Shiau-Wen Liu, Pei-Ling Chi, Chih-Chung Lin, Li-Der Hsiao, Chuen-Mao Yang

**Affiliations:** 1 Department of Physiology and Pharmacology and Health Ageing Research Center, College of Medicine, Chang Gung University, Kwei-San, Tao-Yuan, Taiwan; 2 Department of Anesthetics, Chang Gung Memorial Hospital at Lin-Kou and College of Medicine, Chang Gung University, Kwei-San, Tao-Yuan, Taiwan; University of Nebraska Medical Center, UNITED STATES

## Abstract

Retinal inflammatory diseases induced by cytokines, such as tumor necrosis factor-α (TNF-α) are associated with an up-regulation of intercellular adhesion molecule-1 (ICAM-1) in the retinal pigment epithelial cells (RPECs). Retinal pigment epithelium (RPE) is a monolayer of epithelial cells that forms the outer blood-retinal barrier in the posterior segment of the eye, and is also implicated in the pathology of, such as neovascularization in age-related macular degeneration (AMD). However, the detailed mechanisms of TNF-α-induced ICAM-1 expression are largely unclear in human RPECs. We demonstrated that in RPECs, TNF-α could induce ICAM-1 protein and mRNA expression and promoter activity, and monocyte adhesion. TNF-α-mediated responses were attenuated by pretreatment with the inhibitor of PKCs (Ro318220), PKCδ (Rottlerin), MEK1/2 (U0126), JNK1/2 (SP600125), or AP-1 (Tanshinone IIA) and transfection with siRNA of TNFR1, TRAF2, JNK2, p42, or c-Jun. We showed that TNF-α could stimulate the TNFR1 and TRAF2 complex formation. TNF-α-stimulated JNK1/2 was also reduced by Rottlerin or SP600125. However, Rottlerin had no effect on TNF-α-induced p42/p44 MAPK phosphorylation. We observed that TNF-α induced c-Jun phosphorylation which was inhibited by Rottlerin or SP600125. On the other hand, TNF-α-stimulated ICAM-1 promoter activity was prominently lost in RPECs transfected with the point-mutated AP-1 ICAM-1 promoter plasmid. These results suggest that TNF-α-induced ICAM-1 expression and monocyte adhesion is mediated through a TNFR1/TRAF2/PKCδ/JNK1/2/c-Jun pathway in RPECs. These findings concerning TNF-α-induced ICAM-1 expression in RPECs imply that TNF-α might play an important role in ocular inflammation and diseases.

## Introduction

Age-related macular degeneration (AMD) is the major cause of blindness. The pathogenesis of AMD involves demise of the retinal pigment epithelium (RPE) followed by death of photoreceptors [1]. The RPE is a specialized epithelium at the interface between the neural retina and the choriocapillaris where it forms the outer blood-retinal barrier. Abnormal migration of RPE contributes to a variety of disorders such as proliferative vitreoretinopathy [1].

Tumor necrosis factor-α (TNF-α) is a potent proinflammatory cytokine implicated in tissue damages [2]. The implication of TNF-α in inflammatory responses has been shown to be mediated through up-regulation of inflammatory genes, including intercellular adhesion molecule-1 (ICAM-1) [3]. ICAM-1 (CD54) is an endothelial- and leukocyte-associated transmembrane protein long known for its importance in stabilizing cell-cell interactions and facilitating leukocyte endothelial transmigration. Upon cytokine stimulation, the concentrations of ICAM-1 greatly increase. ICAM-1 can be induced by interleukin-1 (IL-1β) and TNF-α and is expressed by the A549 cells, human airway smooth muscle cells, or human rheumatoid arthritis synovial fibroblasts [3–5]. In addition, AMD and cardiovascular diseases share common risk factors, including ICAM-1 or VCAM-1 up-regulation. However, little is known about the detail mechanisms of signaling pathways involved in TNF-α-induced ICAM-1 expression in human retinal pigment epithelial cells (RPECs). Thus, we used human RPECs to investigate the signaling molecules involved in TNF-α-induced ICAM-1 expression and monocyte adhesion.

Cellular responses to TNF-α are initiated by the type 1 TNFR (TNFR1) and TNFR2 [3]. Most TNF-α biological activity is elicited through the ubiquitous TNFR1, which contains a death domain that fosters protein-protein interactions, particularly with other death-domain proteins [4]. TNF-α activates downstream signaling pathways through these components and induces the expression of various genes that promote immunity and inflammation [4,6]. Protein kinase C (PKC), a large family of serine/threonine kinases, plays multiple essential (patho)physiological roles in various systems by governing various signaling cascades [7]. Depending on the regulatory region, PKCs can be categorized into three groups: the conventional PKCs (-α,-βI,-βII, and -γ) have DAG- and Ca^2+^-binding domains; the novel PKCs (-δ, -ε, -η, -θ) have DAG- but not Ca^2+^-binding domains; the atypical PKCs (-λ/ι, and -ζ) have neither Ca^2+^- nor DAG-binding domains [7]. Moreover, ICAM-1 induction has been shown to be mediated via PKCα [8], PKCδ [9], or PKCθ [10] activation. However, the role of PKCδ in ICAM-1-dependent monocyte adhesion induced by TNF-α is still unclear in RPECs. MAPKs comprise a family of protein-serine/threonine kinases, which are highly conserved in protein structures from unicellular eukaryotic organisms to multicellular organisms, including mammals. These kinases, including p42/p44 MAPK, p38 MAPK, and JNK1/2, are regulated by a phosphor-relay cascade, with a prototype of three protein kinases that sequentially phosphorylate one another. MAPKs transduce extracellular signals into a variety of cellular processes, such as cell proliferation, survival, death, migration, and differentiation [11]. Moreover, we have shown that TNF-α induces ICAM-1 expression via a MAPKs cascade in A549 cells [4]. Huang et al. indicated that TNF-α induces ICAM-1 expression via JNK1/2 and p38 MAPK in human umbilical vein endothelial cells [12]. These results suggested that MAPKs activation also plays a critical role in TNF-α-induced ICAM-1 expression and inflammation.

It has been well established that inflammatory responses following exposure to extracellular stimuli are highly dependent on activation of AP-1 or NF-κB, which plays an important role in the expression of several target genes, such as ICAM-1 [13]. AP-1 is a heterogeneous collection of dimeric transcription factors comprising Jun, Fos, and ATF subunits. Among AP-1 subunits, c-Jun is the most important transcriptional activator in inflammatory status [14]. AP-1 activity is regulated by multiple mechanisms, including phosphorylation by various MAPKs [14]. Nonetheless, the mechanisms underlying TNF-α-stimulated activation of these signaling pathways linking to ICAM-1 expression in RPECs are not completely understood.

In addressing these questions, experiments were undertaken to investigate the mechanisms underlying TNF-α-induced ICAM-1 expression mediated through PKCδ/JNK1/2/c-Jun activation in RPECs. These findings suggest that in RPECs, TNF-α-induced ICAM-1 expression was, at least in part, mediated through a TNFR1/PKCδ/JNK1/2-dependent c-Jun pathway. These results provide new insights into the mechanisms by which TNF-α induced expression of ICAM-1 on RPECs and thus exaggerated the inflammatory responses.

## Materials and Methods

### Materials

Anti-β-actin, anti-ICAM-1, anti-p42, anti-JNK2, anti-TNFR1, anti-c-Jun, and anti-TRAF2 antibodies were from Santa Cruz (Santa Cruz, CA). Anti-phospho-PKCδ, anti-phospho-p42/p44 MAPK, anti-phospho-c-Jun, and anti-phospho-JNK1/2 antibodies were from Cell Signaling (Danver, MA). Ro318220, Rottlerin, Gö6976, U0126, SB202190, SP600125, and Tanshinone IIA were from Biomol (Plymouth Meetings, PA). Anti-TNFR neutralizing antibody, anti-ICAM-1 neutralizing antibody, and TNF-α were from R&D Systems (Minneapolis, MN). All other reagents were from Sigma (St. Louis, MO).

### Cell culture

Human RPECs were from ScienCell Research Lab (San Diego, CA) and cultured in DMEM/F-12 supplemented with 10% FBS and antibiotics (100 U/ml penicillin G, 100 μg/ml streptomycin, and 250 ng/ml fungizone) at 37°C in a humidified 5% CO_2_ atmosphere. When reached confluence, the cells were subcultured to (1 ml/well) 12-well culture plates and (10 ml/dish) 10-cm culture dishes for the measurement of kinases phosphorylation, protein expression, and mRNA accumulation. In these experiments, TNF-α was added to the serum-free medium and incubated for the indicated time intervals. When the inhibitors were used, they were added 1 h before TNF-α treatment. The concentrations of these inhibitors, vehicle DMSO or TNF-α used alone had no toxic effect and change in the cell viability on RPECs, excluded by LDH release test or XTT assay (data not shown).

### Transient transfection with siRNAs

Human siRNAs of scrambled, TNFR1 (HSS110866), TRAF2 (HSS186411), p42 (HSS108535), JNK2 (HSS108550), and Jun (HSS105641) were from Invitrogen (Carlsbad, CA). Human RPECs (passages 4 and 5) were cultured onto 12-well plates. At 70–80% confluence, transient transfection of siRNAs was carried out using Metafectene transfection reagent. Briefly, siRNA (100 nM) was formulated with Metafectene transfection reagent according to the manufacturer’s instruction. The transfection complex was diluted into 400 μl of DMEM/F-12 medium and added directly to the cells. The medium was replaced with serum-free DMEM/F-12 after 24 h. Cells were analyzed at 72 h after transfection by Western blot analysis. The transfection efficiency (approximate 60%) was determined by transfection with EGFP.

### Western blot analysis

Growth-arrested cells were incubated with TNF-α at 37°C for the indicated time intervals. The cells were washed, scraped, collected, and centrifuged at 45000 × *g* at 4°C for 1 h to yield the whole cell extract, as previously described [15]. Samples were denatured, subjected to SDS-PAGE using a 10% running gel, and transferred to nitrocellulose membrane. Membranes were incubated with an anti-ICAM-1, anti-phospho-JNK1/2, anti-phospho-p42/p44 MAPK, anti-phospho-PKCδ, or anti-phospho-c-Jun antibody for 24 h, and then membranes were incubated with an anti-rabbit or anti-mouse horseradish peroxidase antibody for 1 h. The immunoreactive bands were detected by ECL reagents.

### Real-time RT-PCR

Total RNA was extracted using TRIzol reagent. mRNA was reverse-transcribed into cDNA and analyzed by real-time PCR using SYBR Green PCR reagents (Applied Biosystems, Branchburg, NJ) and primers specific for ICAM-1 and GAPDH mRNAs. The levels of ICAM-1 mRNA expression were determined by normalizing to that of GAPDH expression. The primers used were as follows: 5′-GGCCTCAGTCAGTGTGA-3′ (sense) and 5′-AACCCCATTCAGCGTCA-3′ (anti-sense) for ICAM-1; 5′-GCCAAAAGGGTCATCATCTC-3′ (sense) and 5′-GGCCATCCACAGTCTTCT-3′ (anti-sense) for GAPDH.

### Measurement of ICAM-1 luciferase activity

The human ICAM-1 (pIC-339) firefly luciferase was kindly provided by Dr. P. T. van der Saag (Hubrecht Laboratory, Utrecht, The Netherlands). Additionally, the introduction of a mismatched primer mutation into the AP-1 to generate pGL3-ICAM-1 ΔAP-1 was performed, using the following (forward) primer: ΔAP-1: 5′-TTTTTCAAGCTTAGCCTGGCCG-3′. All plasmids were prepared by using QIAGEN plasmid DNA preparation kits. ICAM-1-luc activity was determined as previously described [15] using a luciferase assay system (Promega, Madison, WI).

### Adhesion assay

RPECs were grown to confluence in 6-well plates, incubated with TNF- for 6 h, and then adhesion assays were performed. Briefly, THP-1 cells (human acute monocytic leukemia cell line) were labeled with a fluorescent dye, 10 μM BCECF/AM, at 37°C for 1 h in RPMI-1640 medium (Gibco BRL, Grand Island, NY) and subsequently washed by centrifugation. Confluent RPECs in 6-well plates were incubated with THP-1 cells (2 × 10^6^ cells/ml) at 37°C for 1 h. Non-adherent THP-1 cells were removed and plates were gently washed twice with PBS. The numbers of adherent THP-1 cells were determined by counting four fields per 200X high-power field well using a fluorescence microscope (Zeiss, Axiovert 200M). Experiments were performed in triplicate and repeated at least three times.

### Co-immunoprecipitation assay

Cell lysates containing 1 mg of protein were incubated with 2 μg of an anti-TRAF2 antibody at 4°C for 24 h, and then 10 μl of 50% protein A-agarose beads was added and mixed for 24 h at 4°C. The immunoprecipitates were collected and washed three times with a lysis buffer without Triton X-100. 5X Laemmli buffer was added and subjected to electrophoresis on SDS-PAGE, and then blotted using an anti-TNFR1 or anti-TRAF2 antibody.

### Statistical analysis of data

Data were estimated using a GraphPad Prism Program (GraphPad, San Diego, CA). Quantitative data were expressed as the mean±S.E.M. and analyzed by one-way ANOVA followed with Tukey’s post-hoc test. *P*<0.05 was considered significant.

## Results

### TNF-α induces ICAM-1-dependent monocyte adhesion

**Fig 1:**
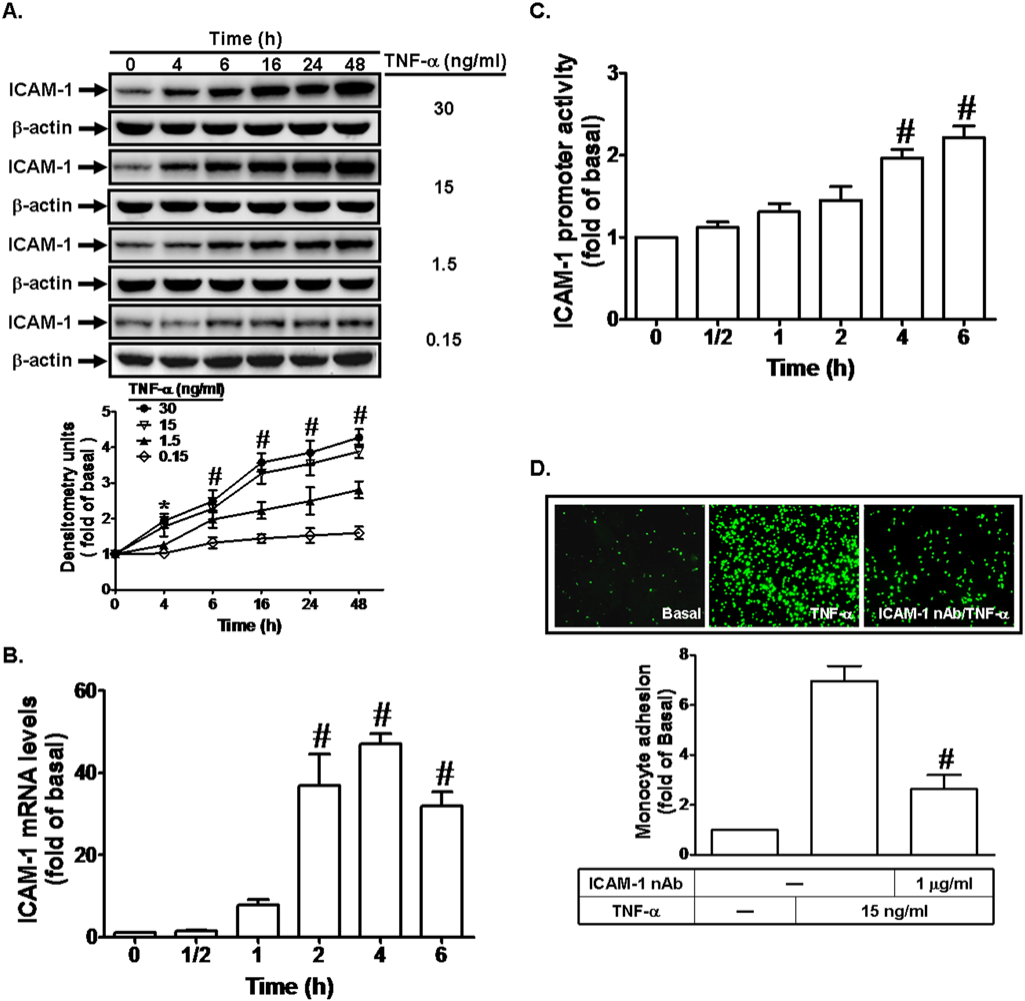
TNF-α induces ICAM-1 expression and monocyte adhesion. (A) Human RPECs were incubated with TNF-α for the indicated time intervals. The protein expression of ICAM-1 was determined by Western blot. (B, C) Cells were incubated with TNF-α (15 ng/ml) for the indicated time intervals. The mRNA levels and promoter activity of ICAM-1 were determined by real-time PCR and promoter assay, respectively. (D) RPECs were pretreated with an ICAM-1 neutralizing antibody for 1 h, and then incubated with TNF-α for 6 h. The THP-1 cells adherence was measured. Data are expressed as mean±S.E.M. of three independent experiments. ^*^ *P*<0.05; ^#^ *P*<0.01, as compared with the cells exposed to vehicle alone (A-C) or TNF-α alone (D).

TNF-α has been shown to induce ICAM-1 expression in various cell types [10,12]. Thus, we first investigated whether TNF-α could induce ICAM-1 expression in RPECs. As shown in Fig. 1A, TNF-α induced ICAM-1 expression in a time- and concentration-dependent manner in these cells. On the other hand, we also found that TNF-α enhanced ICAM-1 mRNA expression and promoter activity in a time-dependent manner with a maximal response within 4 h and 6 h, respectively (Fig. 1B and C). Previous studies have shown that up-regulation of ICAM-1 can increase monocyte adhesion to the cells challenged with cytokines [3]. Thus, we further investigated whether TNF-α-induced ICAM-1 expression could increase monocyte adhesion to RPECs. As shown in Fig. 1D, incubation with TNF-α for 6 h increased THP-1 adhesion to RPECs through ICAM-1 expression, which was attenuated by pretreatment with a neutralizing antibody of ICAM-1. These data suggested that up-regulation of ICAM-1 might play an important functional role in TNF-α-induced monocyte adhesion.

### TNF-α induces ICAM-1 expression via TNFR1/TRAF2

**Fig 2:**
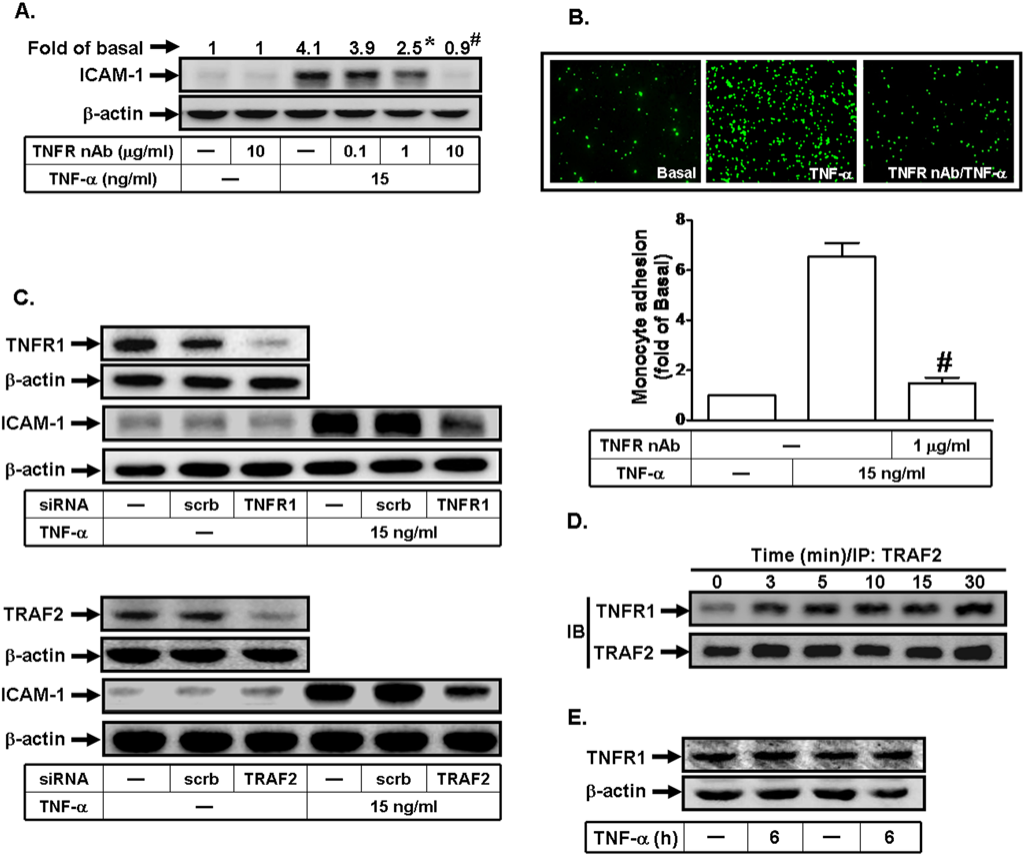
TNF-α-induced ICAM-1 expression is mediated via the formation of a TNFR1/TRAF2 complex. (A) Human RPECs were pretreated with a TNFR neutralizing antibody, and then incubated with TNF-α for 6 h. The protein expression of ICAM-1 was determined by Western blot. (B) RPECs were pretreated with the neutralizing antibody of TNFR for 1 h, and then incubated with TNF-α for 6 h. The THP-1 cells adherence was measured. (C) Cells were transfected with siRNA of scrambled, TNFR1, or TRAF2, and then incubated with TNF-α for 6 h. The protein levels of TNFR1, TRAF2, and ICAM-1 were determined by Western blot. (D) Cells were incubated with TNF-α (15 ng/ml) for various time intervals. The cell lysates were subjected to immunoprecipitation using an anti-TRAF2 antibody, and then the immunoprecipitates were analyzed by Western blot using an anti-TRAF2 or anti-TNFR1 antibody. (E) Cells were treated without or with TNF-α for 6 h. The expression of TNFR1 was determined. Data are expressed as mean±S.E.M. of three independent experiments. ^*^ *P*<0.05; ^#^ *P*<0.01, as compared with the cells exposed to TNF-α alone.

The effects of TNF- are predominantly mediated through TNFR1, which contains a death domain that fosters protein-protein interactions, particularly with other death-domain proteins [2]. To investigate whether TNFR played a key role in TNF-α-mediated ICAM-1 expression, a TNFR neutralizing antibody was used. As shown in Fig. 2A and B, pretreatment with this antibody markedly inhibited TNF-α-induced ICAM-1 expression and monocyte adhesion. Moreover, previous study has demonstrated that TRAF2 is an important adaptor protein in TNF-α-regulated signaling pathways [2]. We further investigated whether TNFR1 and TRAF2 could play key roles in TNF-α-induced ICAM-1 expression. As shown in Fig. 2C, transfection with siRNA of TNFR1 or TRAF2 markedly decreased TNFR1 or TRAF2 total protein expression, and then attenuated TNF-α-induced ICAM-1 expression. Finally, we investigated the physical association between TNFR1 and TRAF2 in TNF-α-induced ICAM-1 expression. Cells were stimulated with TNF-α for the indicated time intervals. The cell lysates were subjected to immunoprecipitation using an anti-TRAF2 antibody, and then the immunoprecipitates were analyzed by Western blot using an anti-TRAF2 or anti-TNFR1 antibody. As shown in Fig. 2D, the protein levels of TNFR1 were time-dependently increased in a TRAF2-immunoprecipitated complex. Moreover, TNF-α had no effect on TNFR1 protein expression in these cells (Fig. 2E). These results suggested that TNF-α triggered the association between TNFR1 and TRAF2 to induce ICAM-1 expression.

### TNF-α induces ICAM-1 expression via a PKCδ

**Fig 3:**
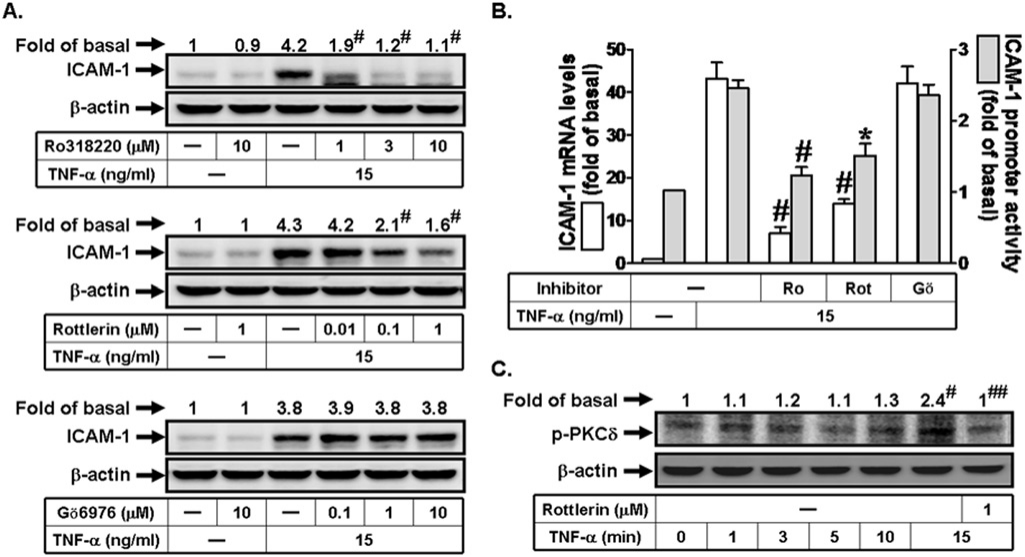
TNF-α induces ICAM-1 expression via a PKCδ signaling in human RPECs. (A) Cells were pretreated with Ro318220, Rottlerin, or Gӧ6976 for 1 h, and then incubated with TNF-α for 6 h. The protein expression of ICAM-1 was determined by Western blot. (B) Cells were pretreated with Ro318220 (10 μM), Rottlerin (1 μM), or Gӧ6976 (10 μM) for 1 h, and then incubated with TNF-α for 4 h or 6 h. The mRNA levels and promoter activity of ICAM-1 were determined by real-time PCR and promoter assay, respectively. (C) Cells were pretreated without or with Rottlerin for 1 h, and then incubated with TNF-α for the indicated time intervals. The levels of phospho-PKCδ were determined by Western blot. Data are expressed as mean±S.E.M. of three independent experiments. ^*^ *P*<0.05; ^#^ *P*<0.01, as compared with the cells exposed to TNF-α alone (A, B). ^#^ *P*<0.01, as compared with the cells exposed to vehicle alone (C). ^##^ *P*<0.01, as compared with the cells exposed to TNF-α alone (C).

Some studies have indicated that the expression of ICAM-1 is mediated through the activation of PKCs [8,10]. Thus, we investigated the roles of PKCs in TNF-α-induced ICAM-1 expression. As shown in Fig. 3A and B, pretreatment with the inhibitor of non-selective PKC (Ro318220) or PKCδ (Rottlerin), but not PKCα/β (Gӧ6976) markedly attenuated TNF-α-induced ICAM-1 protein expression, mRNA levels, and promoter activity. In this study, we observed that TNF-α could stimulate PKCδ phosphorylation in a time-dependent manner, which was inhibited by Rottlerin (Fig. 3C). These data demonstrated that PKCδ plays an important role in TNF-α-induced ICAM-1 expression in RPECs.

### TNF-α induces ICAM-1 expression via a PKCδ/JNK1/2 signaling

**Fig 4:**
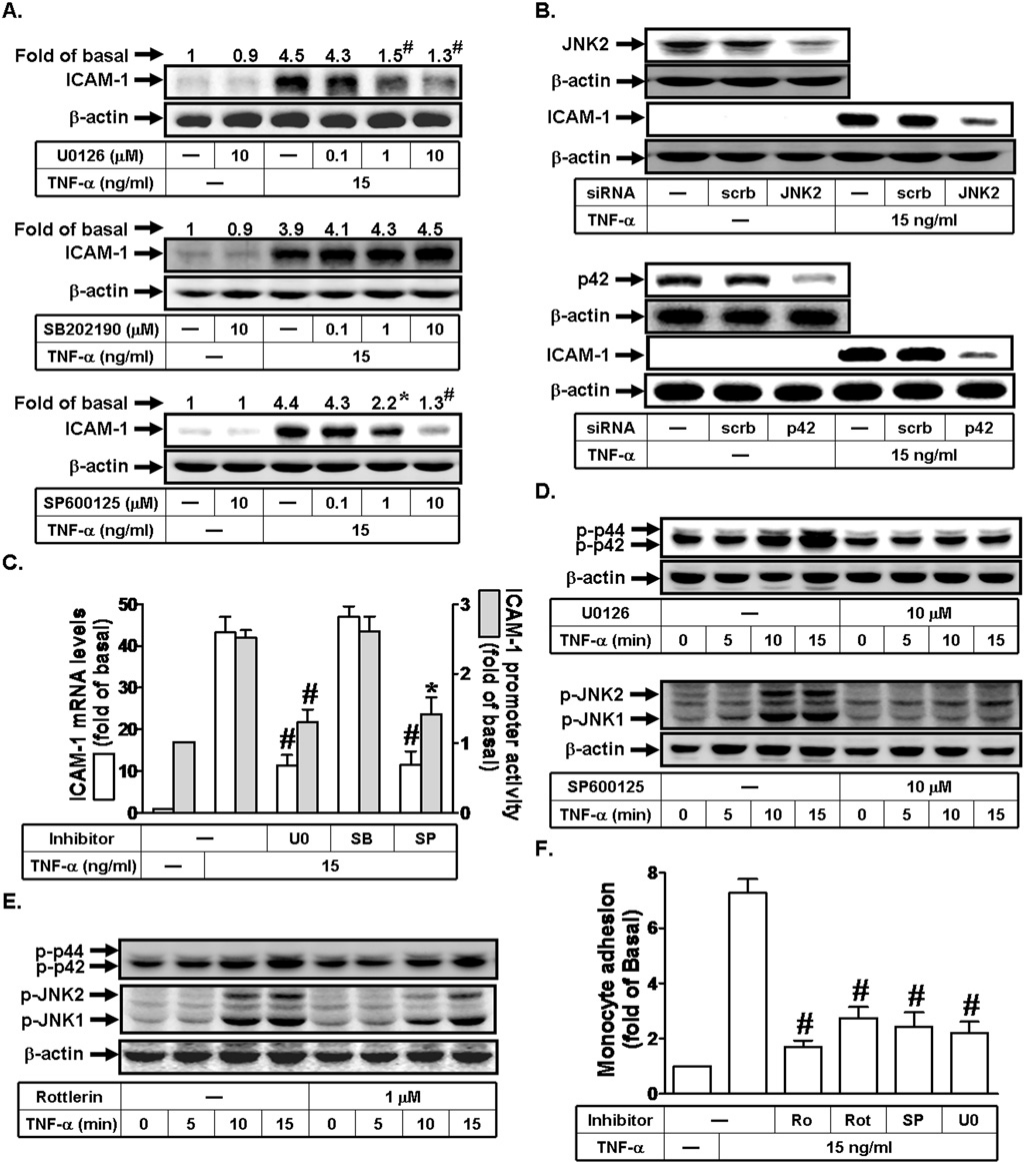
TNF-α induces ICAM-1 expression via a PKCδ/JNK1/2 signaling in human RPECs. (A) Cells were pretreated with U0126, SP600125, or SB202190 for 1 h, and then incubated with TNF-α for 6 h. The protein expression of ICAM-1 was determined by Western blot. (B) Cells were transfected with siRNA of scrambled, JNK2, or p42, and then incubated with TNF-α for 6 h. The protein levels of p42, JNK2, and ICAM-1 were determined by Western blot. (C) Cells were pretreated with U0126 (10 μM), SP600125 (10 μM), or SB202190 (10 μM) for 1 h, and then incubated with TNF-α for 4 h or 6 h. The mRNA levels and promoter activity of ICAM-1 were determined by real-time PCR and promoter assay, respectively. (D) Cells were pretreated without or with U0126 or SP600125 for 1 h, and then incubated with TNF-α for the indicated times. The levels of phospho-p42/p44 MAPK and phospho-JNK1/2 were determined by Western blot. (E) Cells were pretreated without or with Rottlerin for 1 h, and then incubated with TNF-α for the indicated time intervals. The levels of phospho-p42/p44 MAPK and phospho-JNK1/2 were determined by Western blot. (F) RPECs were pretreated with Ro318220, Rottlerin, SP600125, or U0126 for 1 h, and then incubated with TNF-α for 6 h. The THP-1 cells adherence was measured. Data are expressed as mean±S.E.M. of three independent experiments. ^*^ *P*<0.05; ^#^ *P*<0.01, as compared with the cells exposed to TNF-α alone.

MAPKs integrate signals from numerous receptors and translate these signals into cellular functions. MAPKs are critical for metabolism, migration, production of pro-inflammatory mediators, survival and differentiation. Several reports have suggested that activation of MAPKs leads to the expression of inflammatory genes, including ICAM-1 [4,12]. In this study, we used the inhibitor of MEK1/2 (U0126), p38 MAPK (SB202190), or JNK1/2 (SP600125) to investigate the role of p42/p44 MAPK, JNK1/2, or p38 MAPK in TNF-α-induced ICAM-1 expression. As shown in Fig. 4A, pretreatment with U0126 or SP600125, but not SB202190 markedly inhibited TNF-α-induced ICAM-1 protein expression, mRNA levels, and promoter activity. We further used siRNA of JNK2 or p42 to confirm the roles of JNK1/2 and p42/p44 MAPK in TNF-α-mediated responses. As shown in Fig. 4B, transfection with JNK2 or p42 siRNA attenuated TNF-α-induced ICAM-1 expression in these cells. On the other hand, U0126 and SP600125 also reduced TNF-α-induced ICAM-1 mRNA levels and promoter activity (Fig. 4C). Here, we also observed that TNF-α could stimulate p42/p44 MAPK and JNK1/2 phosphorylation in a time-dependent manner, which were inhibited by U0126 and SP600125, respectively (Fig. 4D). Next, we investigated the relationship between PKCδ and MAPKs in TNF-α-mediated responses. As shown in Fig. 4E, pretreatment with Rottlerin markedly reduced TNF-α-stimulated JNK1/2, but not p42/p44 MAPK phosphorylation. Finally, we found that increase in monocyte adhesion to RPECs challenged with TNF-α was attenuated by Rottlerin, Ro318220, SP600125, or U0126 (Fig. 4F). Thus, we suggested that TNF-α-induced ICAM-1 expression is mediated through a PKCδ/JNK1/2 pathway.

### TNF-α induces ICAM-1 expression via a c-Jun

**Fig 5:**
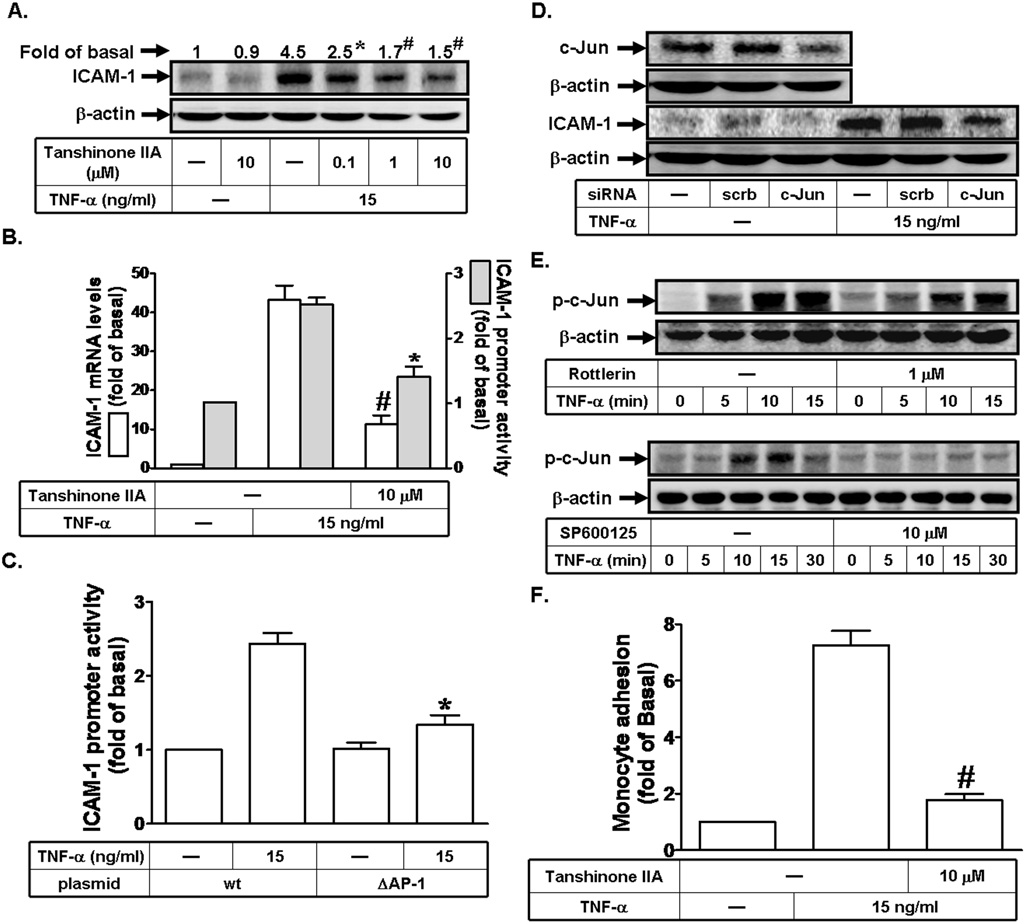
TNF-α induces ICAM-1 expression through a c-Jun pathway. (A) Human RPECs were pretreated with Tanshinone IIA for 1 h, and then incubated with TNF-α for 6 h. The protein expression of ICAM-1 was determined by Western blot. (B) Cells were pretreated with Tanshinone IIA (10 μM) for 1 h, and then incubated with TNF-α for 4 h or 6 h. The mRNA levels and promoter activity of ICAM-1 were determined by real-time PCR and promoter assay, respectively. (C) Cells were transfected with wild-type ICAM-1 promoter-luciferaase or mutant ICAM-1 promoter-luciferaase (ΔAP-1), and then incubated with TNF-α (15 ng/ml) for 6 h. The ICAM-1 promoter activity was determined in the cell lysates. (D) Cells were transfected with siRNA of scrambled or c-Jun, and then incubated with TNF-α for 6 h. The protein levels of c-Jun and ICAM-1 were determined by Western blot. (E) Cells were pretreated without or with Rottlerin or SP600125 for 1 h, and then incubated with TNF-α for the indicated time intervals. The levels of phospho-c-Jun were determined by Western blot. (F) RPECs were pretreated with Tanshinone IIA for 1 h, and then incubated with TNF-α for 6 h. The THP-1 cells adherence was measured. Data are expressed as mean±S.E.M. of three independent experiments. ^*^ *P*<0.05; ^#^ *P*<0.01, as compared with the cells exposed to TNF-α alone (A, B, F). ^*^ *P*<0.05, as compared with the cells transfected with wild-type ICAM-1 promoter-luciferaase (C).

It has been well established that inflammatory responses following exposure to extracellular stimuli are highly dependent on activation of AP-1, which plays an important role in the expression of several target genes, such as ICAM-1 [13]. Among AP-1 subunits, c-Jun is the most important transcriptional activator in inflammatory status [14]. Therefore, we first determined whether TNF-α-induced ICAM-1 expression was mediated through AP-1 in RPECs. As shown in Fig. 5A and B, pretreatment with Tanshinone IIA (an AP-1 inhibitor) markedly reduced TNF-α-induced ICAM-1 expression, mRNA levels, and promoter activity. To further confirm the role of AP-1 in TNF-α-mediated ICAM-1 promoter induction, a point-mutated AP-1 ICAM-1 promoter construct was used. As shown in Fig. 5C, TNF-α-stimulated ICAM-1 promoter activity was prominently lost in RPECs transfected with the point-mutated AP-1 ICAM-1 promoter plasmid. To confirm the role of AP-1 in TNF-α-induced ICAM-1 expression, c-Jun siRNA was used. We found that transfection with c-Jun siRNA significantly reduced the protein expression of c-Jun, and then inhibited TNF-α-induced ICAM-1 expression (Fig. 5D). Next, we found that TNF-α markedly stimulated c-Jun phosphorylation, which was inhibited by Rottlerin or SP600125 (Fig. 5E). Finally, we observed that pretreatment with Tanshinone IIA reduced monocyte adhesion to RPECs challenged with TNF-α (Fig. 5F). Taken together, we suggested that TNF-α-induced ICAM-1 expression is mediated through a PKCδ/JNK1/2/c-Jun signaling pathway.

## Discussion

AMD is a blinding disease, affecting millions of elderly individuals. In the most severe manifestation of AMD, blood vessels grow from the choroid into the subretinal pigment epithelial (subRPE) and subretinal space, leading to severe visual loss that can be sudden and permanent [16]. Retinal inflammatory diseases induced by cytokines, such as TNF-α and IL-1β are associated with an up-regulation in the expression of ICAM-1 in cells within the retina. TNF-α has been shown to regulate the expression of ICAM-1 through various signaling pathways, such as c-Src, NADPH oxidase/ROS, NF-kB, ATF2, and STAT3 in various cell types [3,4]. However, the mechanisms underlying TNF-α-induced ICAM-1 expression in RPECs remain largely unknown. In this study, we applied Western blot, real-time PCR, promoter assay, selective pharmacological inhibitors, and siRNAs to investigate the mechanisms of TNF-α-induced ICAM-1 expression in these cells. We showed that TNF-α-induced ICAM-1 expression was mediated through a TNFR1/TRAF2/PKCδ/JNK1/2-dependent c-Jun signaling pathway in human RPECs.

ICAM-1 (CD54) is an endothelial- and leukocyte-associated transmembrane protein long known for its importance in stabilizing cell-cell interactions and facilitating leukocyte endothelial transmigration. ICAM-1 plays a role in pathological processes including inflammation, arthritis, cardiovascular diseases, and eye diseases. Accumulating evidence demonstrates that TNF-α may activate downstream protein kinases leading to the expression of inflammatory proteins [3,4]. All known responses to TNF are triggered by binding to one of two distinct receptors, designated as TNFR1 and TNFR2 [3]. However, based on cell culture experiments and studies with receptor knockout mice, both the proinflammatory and the programmed cell death pathways that are activated by TNF, and associated with tissue injury, are largely mediated through TNFR1 [3,17]. Indeed, in RPECs, we established that TNF-α induced ICAM-1 expression via a TNFR1-dependent pathway. In addition, we also demonstrated that TNF-α induced monocyte adhesion via an ICAM-1-dependent signaling. Moreover, previous study has demonstrated that TRAF2 is an important adaptor protein in TNF-α-mediated signaling pathways [2]. We also observed that TNF-α-induced ICAM-1 expression was reduced via TRAF2 siRNA. We further investigated the physical association of TNFR1 and TRAF2 in TNF-α-induced ICAM-1 expression. Our results established that TNF-α induced the formation of a TNFR1/TRAF2 complex leading to ICAM-1 expression in human RPECs.

PKC represents a family of more than 11 phospholipid-dependent Ser/Thr kinases that are involved in a variety of pathways that regulate cell growth, death, and stress responsiveness [7]. PKC isoforms are divided into three categories according to the cofactors that are required for optimal phospholipid-dependent catalytic activity [7]. Moreover, ICAM-1 induction has been shown to be mediated via PKCα [8], PKCδ [9], or PKCθ [10] activation. In our study, we found that the inhibition of PKCα/β had no effect on TNF-α-induced ICAM-1 expression in RPECs. However, PKCδ inhibition by Rottlerin could reduce TNF-α-induced ICAM-1 protein levels and mRNA expression, suggesting that PKCδ activation plays a key role in mediating TNF-α-enhanced ICAM-1 expression in RPECs. In the future, we will investigate whether ICAM-1 induction is mediated via other PKC isozymes in TNF-α-stimulated RPECs.

The MAPKs family consists of three major members: ERK1/2, p38 MAPK, and JNK1/2. MAPKs are important intracellular signalings and play critical roles in inflammatory responses. Previous study has indicated that TNF-α induced ICAM-1 expression and monocyte adhesion via MAPKs in A549 cells [4]. In addition, Huang et al. indicated that TNF-α induced ICAM-1 expression via JNK1/2 and p38 MAPK activation in human umbilical vein endothelial cells [12]. Recently, we also showed that IL-1β induced ICAM-1 expression via p42/p44 MAPK and JNK1/2 in human rheumatoid arthritis synovial fibroblasts [5]. In human RPECs, we observed that both p42/p44 MAPK and JNK1/2, but not p38 MAPK were involved in TNF-α-induced ICAM-1 expression. We further investigated the relationship between PKCδ and p42/p44 MAPK or JNK1/2 in TNF-α-stimulated RPECs. However, we found that PKCδ plays a critical role in mediating TNF-α-induced JNK1/2, but not p42/p44 MAPK activation. In the future, we will investigate the detail mechanisms involved in TNF-α-mediated p42/p44 MAPK activation in RPECs.

It has been well established that inflammatory responses following exposure to extracellular stimuli are highly dependent on activation of AP-1 or NF-kB, which plays an important role in the expression of several target genes [18]. AP-1 is a sequence-specific transcriptional activator mainly composed of members of the Jun and Fos families. The ICAM-1 promoter has been shown to contain several binding sequences for various transcription factors, including AP-1 [9]. These studies suggest that AP-1 plays a critical role in the regulation of ICAM-1 expression in the development of the inflammatory responses. Our data showed that TNF-α-induced ICAM-1 protein and mRNA expression and monocyte adhesion was significantly abolished by pretreatment with an AP-1 inhibitor (Tanshinone IIA) or transfection with c-Jun siRNA, implying that c-Jun was involved in TNF-α-induced ICAM-1 expression in human RPECs. Our data further showed that pretreatment with a JNK1/2 inhibitor (SP600125) attenuated c-Jun phosphorylation, consistent with a recent report showing that phorbol ester stimulated AP-1 activation via JNK1/2 in human breast epithelial cells [19]. In addition, pretreatment of RPECs with Rottlerin significantly inhibited c-Jun phosphorylation stimulated by TNF-α. These findings suggested that in RPECs, TNF-α-induced ICAM-1 expression and monocyte adhesion is mediated via a PKCδ/JNK1/2/c-Jun signaling pathway. On the other hand, NF-kB is a ubiquitous transcription factor that, through target genes, regulates key processes such as inflammation, apoptosis, stress response, wound healing, angiogenesis, and lymphangiogenesis. Numerous recent studies have investigated NF-kB in the context of ocular surface disorders, including chemical injury, ultraviolet radiation-induced injury, microbial infections, allergic eye diseases, dry eye, pterygium, and corneal graft rejection [20]. NF-kB has been shown to regulate ICAM-1 induction [3]. Indeed, in human RPECs, NF-kB inhibition also reduced TNF-α-induced ICAM-1 expression (unpublished data). In the future, we will investigate the detailed mechanisms of TNF-α-mediated NF-kB activation in these cells.

**Fig 6:**
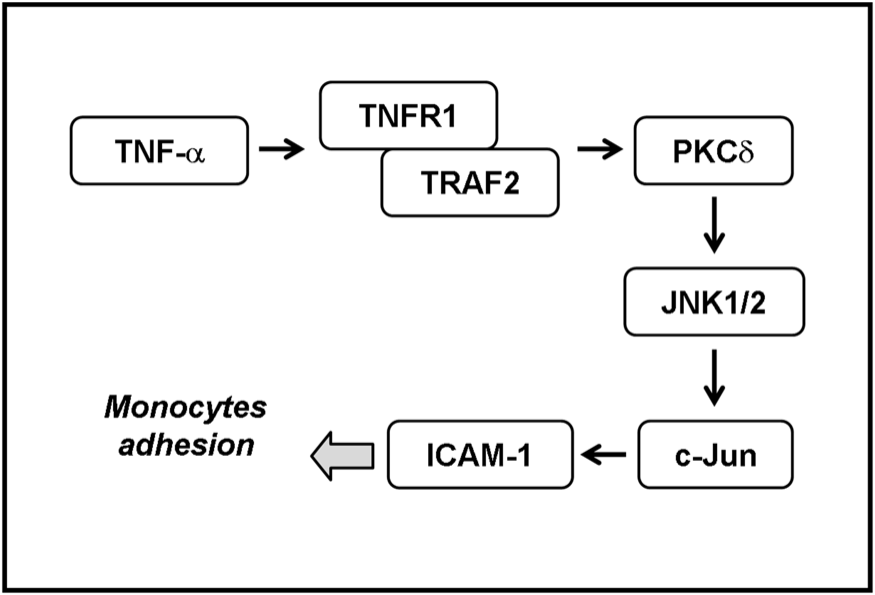
Schematic representation of the signaling pathways involved in the TNF-α-induced ICAM-1 expression in RPECs. TNF-α-induced ICAM-1 expression and monocyte adhesion are mediated through a TNFR1/TRAF2/PKCδ/JNK1/2/c-Jun signaling pathway in human RPECs.

Base on the observation from literatures and our findings, Fig. 6 reveals a model for the signaling mechanisms implicated in TNF-α-induced ICAM-1 expression in human RPECs. These findings concerning TNF-α-induced ICAM-1 expression and monocyte adhesion imply that TNF-α might play an important role in ocular inflammatory diseases, mediated through a TNFR1/TRAF2/PKCδ/JNK1/2/c-Jun signaling pathway in human RPECs. These results indicated a role for human RPECs, in addition to their physiological function, as inflammatory cells involved in the production of chemical mediators which may contribute to the inflammatory responses seen in various ocular diseases.
